# Detection and preliminary evaluation of circulating tumor cells in the peripheral blood of patients with eight types of cancer using a telomerase-specific adenovirus

**DOI:** 10.3892/or.2014.3436

**Published:** 2014-08-22

**Authors:** MINA YABUSAKI, JUN SATO, ATSUSHI KOHYAMA, TAKASHI KOJIMA, DAISUKE NOBUOKA, TOSHIAKI YOSHIKAWA, YU SAWADA, KATSUHIRO MURAKAMI, KEIGO GOHDA, TAKATSUGU OKEGAWA, MASARU NAKAMURA, KIYOSHI TAKAMATSU, MASAAKI ITO, KAZUHIRO KANEKO, TETSUYA NAKATSURA

**Affiliations:** 1 Central Research Laboratories, Sysmex Corporation, Kobe, Hyogo, Japan; 2 Division of Cancer Immunotherapy, Exploratory Oncology Research and Clinical Trial Center, National Cancer Center, Kashiwa, Chiba, Japan; 3 Division of Colorectal and Pelvic Surgery, National Cancer Center Hospital East, Kashiwanoha, Kashiwa, Chiba, Japan; 4 Department of Gastroenterology, Endoscopy Division, National Cancer Center Hospital East, Kashiwa, Chiba, Japan; 5 Department of Gastroenterological Surgery, Okayama University, Okayama, Japan; 6 Department of Urology, Kyorin University School of Medicine, Mitaka, Tokyo, Japan; 7 Department of Obstetrics and Gynecology, Tokyo Dental College, Ichikawa General Hospital, Ichikawa, Chiba, Japan

**Keywords:** adenovirus, circulating tumor cells, OBP-401, telomerase

## Abstract

We developed a detection method for circulating tumor cells (CTCs) using the telomerase-specific adenovirus OBP-401. This recombinant virus has a telomerase promoter at the 5′-end of the viral genome and GFP at the 3′-end. To date, CTC enumeration using OBP-401 has shown prognostic impact for gastric and small cell lung cancer patients. In the present study, peripheral blood samples from patients with eight types of cancer, including some cancers previously untested with OBP-401 (i.e., esophagus, pancreas, and prostate cancers) were subjected to this method in order to evaluate its versatility. It was recently discovered that some white blood cells (WBCs) false-positively react with OBP-401. Although anti-CD45 antibodies can absorb these adverse cells from peripheral blood, the simplicity of the OBP-401 method would be diminished by the introduction of antibody treatment. Therefore, we evaluated another approach to minimize the false positivity of WBCs. Seven anti-CD antibodies were employed to stain the species of WBCs that false-positively reacted with OBP-401. We revealed that the false-positively reacted WBCs were monocytes in the peripheral blood of both healthy subjects and cancer patients. Based on a size distribution analysis of the GFP-positive monocytes, the size criterion for CTCs using OBP-401 was defined to be a cellular diameter >8.4 μm. In total, 43% of 86 cancer patients examined in the present study were CTC-positive using this definition. CTCs were enumerated from peripheral blood samples collected from patients with each of the eight types of cancer; the detectability of CTCs for esophagus, pancreas and prostate cancers by the OBP-401 method was confirmed for the first time in the present study. However, no clear correlation between CTC positivity and the clinical characteristics of patients with any type of cancer was observed because of the small number of patients with each type of cancer. An additional clinical study will be conducted to confirm the clinical meaning of CTCs enumerated by OBP-401.

## Introduction

Circulating tumor cells (CTCs) in peripheral blood are becoming a promising sign for use in predicting the prognosis and/or treatment effects in patients with various types of cancer (1). To capture CTCs, various methods have been developed (1). The most widely used method relies on anti-EpCAM antibodies (2). This method has been approved by the FDA for determining the prognosis of breast, prostate or colon cancer (3–6).

Recently, we developed a novel CTC-detection method using a recombinant telomerase-specific adenovirus, OBP-401, which carries a telomerase promoter at the 5′-end of the viral genome and *GFP* at the 3′-end (7). After the viral infection of blood samples from cancer patients, GFP fluorescence in the CTCs can be monitored due to the positivity of telomerase activity in various types of tumor cells (8–10). In fact, several types of tumor cells have so far been detected using OBP-401 from breast, gastric, colon, liver, gynecological and small cell lung cancers (11–15). In particular, for gastric and small cell lung cancers, CTC positivity in patients as determined by OBP-401 was reported as a prognostic risk, indicating the clinical utility of OBP-401 (13,14). In the present study, we strove to detect and preliminary evaluate CTCs in the peripheral blood of patients with unexamined cancers by OBP-401 to estimate the versatility of this method.

However, a recent report showed that OBP-401 infects white blood cells (WBCs), leading to false positivity (15). To eliminate the adverse effect of WBC infection by OBP-401, they introduced a procedure for WBC absorption using anti-CD45 antibodies and succeeded in enumerating CTCs (15). In the present study, we minimized the effect of WBC infection with OBP-401 by clarifying the false-positive WBCs using anti-CD antibody staining experiments and size selection between WBCs and CTCs, which would be a much simpler method since no antibody absorption process is involved. The size selection was also used for the direct enrichment of CTCs by filtration method (16,17). Using the criteria derived from our staining and selection methods, CTCs in peripheral blood samples from patients with eight different types of cancer were enumerated by OBP-401.

## Materials and methods

### Patients and healthy subjects

A total of 86 patients who were treated at the National Cancer Center Hospital East (Kashiwa, Japan), the Kyorin University Hospital (Mitaka, Japan), and the Tokyo Dental College Ichikawa General Hospital (Ichikawa, Japan) were recruited for this study. The inclusion criteria were: i) signed informed consent and ii) newly diagnosed esophageal, stomach, colon, liver, pancreatic, prostate, endometrial or cervical cancer without preoperative chemotherapy or radiotherapy. The disease stage was determined according to the sixth edition of the TNM classification of the International Union Against Cancer. We also assessed six healthy subjects. All the patients and healthy subjects provided written informed consent. The institutional review boards of all the institutes approved the experiments undertaken in the present study.

### Virus

OBP-401, a telomerase-specific, replication-selective adenoviral agent in which the TERT promoter element drives the expression of *EIA* and *EIB*, and into which *GFP* is integrated, was used (7). Viral samples were stored at −80°C.

### Detection of CTCs by the OBP-401 assay

The details of the assay were previously described (12). Blood samples were collected before surgery or chemotherapy. Samples were drawn into citrate, phosphate and dextrose-containing tubes and stored at 4°C until assayed. A sample was centrifuged for 5 min at 540 × g, and the plasma phase was removed. The cells were then washed four times with phosphate-buffered saline (PBS) and twice with Roswell Park Memorial Institute medium. The sample was infected with 4×10^8^ plaque-forming units (PFU) of OBP-401 virus and incubated in the medium for 24 h at 37°C. To inactivate OBP-401, the cells were fixed with an equal amount of 4% paraformaldehyde for 20 min at room temperature. The sample was treated with a surface-active agent (Emalgen 2025 G; Kao Chemicals, Tokyo, Japan) for 10 min at 40°C to degrade red blood cells. Finally, 7.5 ml of blood was used to create two glass slides for microscopic analysis. Images were captured and were recorded with an inverted microscope (IX71; Olympus, Tokyo, Japan). Original software was used to count the number of GFP-expressing cells and measure the cell size and GFP intensity.

### Immunocytochemistry for GFP-positive cells

Blood samples were centrifuged and washed four times with PBS. The buffy coat was collected from the blood with a pipette. Virus infection and fixation followed the procedure described above. The cells were stained with DAPI, PerCP-Cy5.5 anti-human CD45 (304027; BioLegend, San Diego, CA, USA); and one of the following antibodies: PE anti-human CD2 (309207), PE anti-human CD13 (301703), PE anti-human CD14 (301805), PE anti-human CD15 (301905), PE anti-human CD19 (302207), PE anti-human CD45 (304008) (all from BioLegend), and PE anti-human CD203c (130092243; Miltenyi Biotec Inc., San Diego, CA, USA).

### Statistical analysis

The correlation of CTC positivity with clinical variables was analyzed by the Chi-square test using MedCalc statistical software version 13.0 (MedCalc Software bvba, Ostend, Belgium, 2014).

## Results

### Most GFP(+) WBCs from the healthy subjects and patients were monocytes

**Figure 1:**
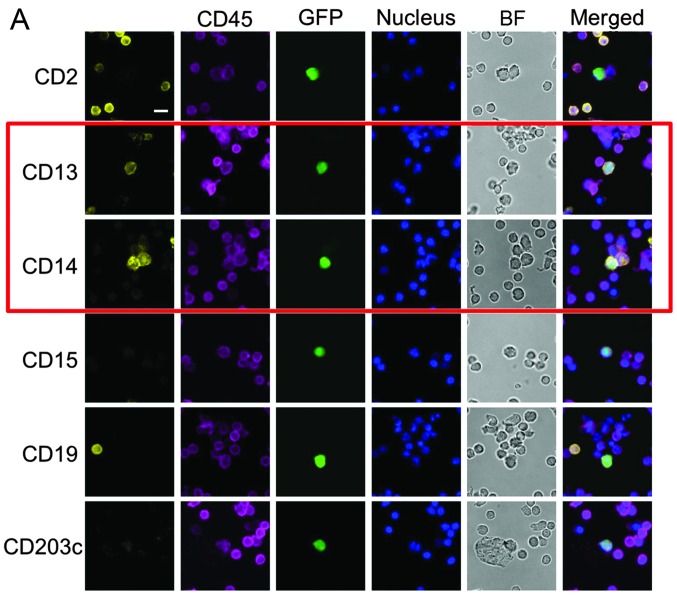

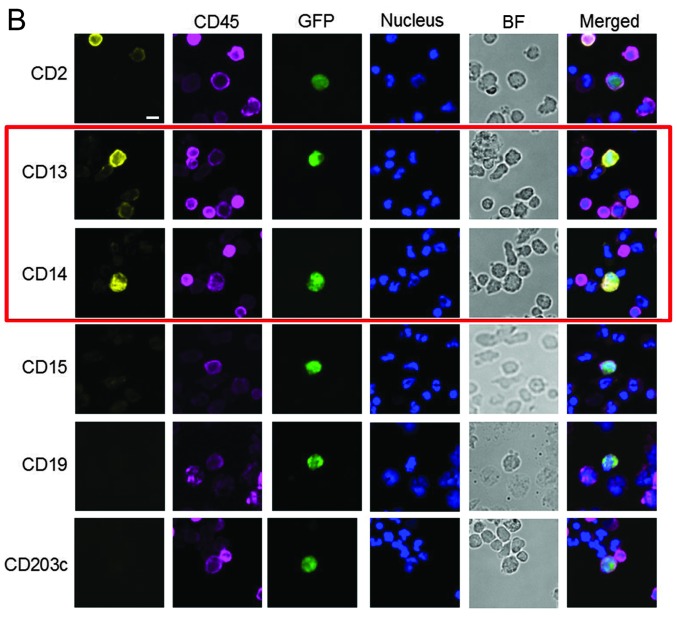
(A) Anti-CD antibody staining for peripheral blood samples shows that the GFP(+) cells in the healthy subjects were monocytes. Nuclei were stained with DAPI. CD45 was stained using PerCP-Cy5.5-conjugated antibody. All other CD antigens were stained using PE-conjugated antibodies. (B) CD45(+)/GFP(+) cells in peripheral blood samples from the cancer patients confirmed that the monocytes showed positivity. Nuclei were stained with DAPI. CD45 was stained using PerCP-Cy5.5-conjugated antibody All other CD antigens were stained using PE-conjugated antibodies. BF, bright field. Scale bar, 10 μm.

First, peripheral blood samples from six healthy subjects were treated with OBP-401, and fluorescent signals from a few dozen cells from each sample were detected. The GFP-positive cells from the healthy subjects were subjected to identification of the cellular species based on staining with anti-CD antibodies. The seven anti-CD antibodies used in the present study covered the detection of various types of WBCs, from lymphocytes to granulocytes (CD2, CD13, CD14, CD15, CD19, CD45 and CD203c). Double-positive signals for the anti-GFP and anti-CD antibodies were obtained using CD13 or CD14 staining (Fig. 1A). The positivity rate for each anti-CD antibody is shown in Table I. All of the GFP-positive cells that were CD13- or CD14-positive showed a positive signal by CD45 staining, indicating that the CD13(+)/GFP(+) and CD14(+)/GFP(+) cells were monocytes.

To confirm this observation in cancer patients, peripheral blood samples from two digestive cancer patients were treated using the same anti-CD antibody classification system as in the experiments with healthy subjects. Again, high positivity was obtained by CD13 or CD14 staining among the CD45(+)/GFP(+) cells (Fig. 1B and Table II). These results clearly demonstrate that the GFP-positive WBCs were monocytes.

### CTC criteria defined to exclude monocytes

**Figure 2:**
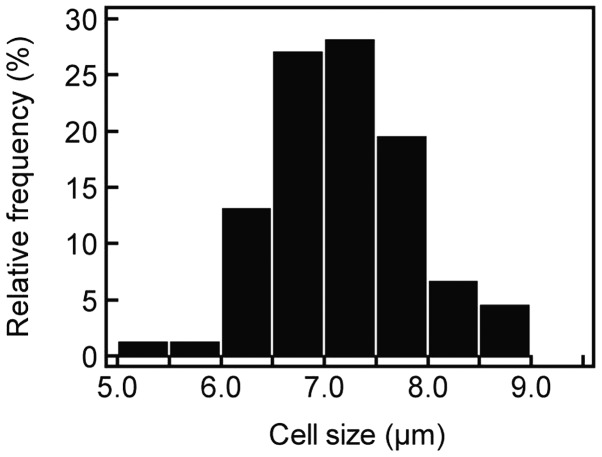
The monocytes from healthy subjects followed a normal distribution of cellular diameter.

**Figure 3:**
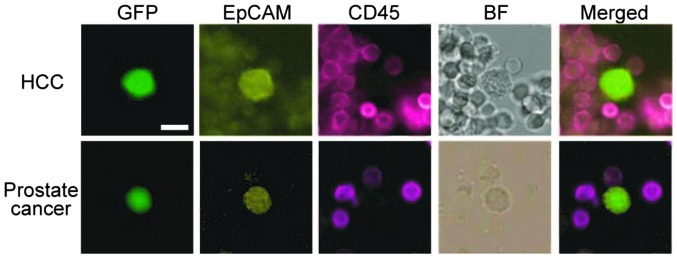
The CTCs observed in the liver and prostate cancer patients showed EpCAM positivity and CD45 negativity. EpCAM was using Pacific Blue-conjugated antibody. CD45 was stained using PE-conjugated antibody. BF, bright field. CTCs, irculating tumor cells. Scale bar, 10 μm.

In general, the cellular diameter of CTCs is greater than that of WBCs (17–19). Therefore, we hypothesized that the GFP(+) monocytes could be distinguished from other GFP(+) cells on the basis of size differences. The cellular diameter of the GFP(+) monocytes in the blood samples from the healthy subjects exhibited a normal distribution (Fig. 2). The average and standard deviation (SD) of the monocyte distribution were 7.18 and 0.65 μm, respectively. Using these data, we set the criteria for the exclusion of monocytes among GFP(+) cells by a 95% confidence interval (i.e., average plus 2× SD) to be 8.4 μm. Using this size criterion, CTCs in the present study were defined as GFP(+) cells with a >8.4 μm cellular diameter. CTCs having EpCAM positivity as well as CD45 negativity are shown from liver and prostate cancer patients in Fig. 3.

### Analysis of peripheral blood samples from patients with eight types of cancer

Based on the criteria for CTC detection, peripheral blood samples from patients with eight types of cancer were analyzed: cancers of the esophagus, stomach, colon, pancreas, liver, endometrium, cervix and prostate. The patient demographic and clinical characteristics are summarized in Table III. All of the types of cancer examined using OBP-401 in the present study exhibited CTC deployment in peripheral blood samples. In total, 43% (37 of 86) of the patients with the eight types of cancer showed at least one CTC. Clinical stage and other clinical parameters were not correlated with CTC positivity (Table IV).

## Discussion

In previous studies, it was reported that some blood cells in healthy samples reacted with OBP-401, leading to false positivity for CTC detection (20). Recently, those GFP-positive cells were determined to be WBCs by anti-CD45 antibody staining (15). In that study, a method was developed to remove WBCs that could lead to false-positive readings by anti-CD45 antibody absorption to specifically enumerate CTCs in blood samples (15). This method effectively minimizes false positivity but decreases the simplicity of CTC detection using the OBP-401 methodology (11).

In the present study, we utilized another approach to minimize false positivity. This approach was based on size selection using the difference in cellular diameter between monocytes and CTCs (17–19). The blood cells false-positively reacted by OBP-401 were determined to be monocytes using anti-CD antibody staining. Using the size criterion of 8.4 μm for GFP(+) monocytes, 95.5% of the monocytes among GFP(+) cells can theoretically be eliminated. In fact, monocytes are known to have telomerase activity and to express Coxsackie/adenovirus receptor, which is necessary for the adenovirus to infect the cells (21,22).

By use of this criterion, CTCs from patients with eight types of cancer were successfully enumerated. This is the first study to monitor CTCs using the OBP-401 method in peripheral blood samples from patients with cancers of the esophagus, pancreas and prostate. The biggest advantage of our method is that the detection of cancer cells is based on hTERT activity, which is ubiquitously observed in various types of cancer (8–10). The versatility of CTC enumeration by OBP-401 may overcome the drawback of an immunocapturing method such as EpCAM since it is known that epithelial features of cancer cells are lost and changed to mesenchymal ones during metastasis or invasion (i.e., epithelial-mesenchymal transition).

In the present study, no clearly significant clinical relationship was observed between CTC positivity and clinical characteristics because of the limited number of patients with each type of cancer. However, as previously shown in reports on breast, gastric and small cell lung cancers, the accumulation of a certain number of clinical data will allow the clinical significance of CTC positivity rates to be demonstrated. Further clinical study is thus currently planned to prove our concept.

## Figures and Tables

**Table I:** Anti-CD antibody staining of peripheral blood samples from healthy subjects.

CD antigens	No. of GFP(+) cells	No. of CD(+) among GFP(+) cells	CD(+) rates among GFP(+) cells (%)
CD2	89	2	2.2
CD13	54	52	96
CD14	125	87	70
CD15	83	2	2.4
CD19	70	0	0.0
CD45	76	76	100
CD203c	38	0	0.0

**Table II:** Anti-CD antibody staining of peripheral blood samples from two cancer patients.

CD antigens	No. of GFP(+) cells	No. of CD(+) among GFP(+) cells	CD(+) rates among GFP(+) cells (%)
CD2	20	0	0.0
CD13	17	13	76
CD14	23	8	35
CD15	19	0	0.0
CD19	16	0	0.0
CD45	116	116	100
CD203c	21	0	0.0

**Table III:** Patient demographic and clinical characteristics by type of cancer.

	Esophagus (n=12)	Stomach (n=11)	Colon (n=18)	Pancreas (n=12)	Liver (n=11)	Endometrium (n=4)	Cervix (n=8)	Prostate (n=10)
Age in years [mean (SD)]	68.2 (5.8)	59.5 (11.1)	60.6 (14.7)	69.9 (8.9)	70.4 (7.3)	58.5 (6.8)	51.5 (14.3)	77.1 (8.0)
Gender, n (%)								
Male	12 (100)	9 (82)	10 (56)	10 (83)	6 (55)	0 (0)	0 (0)	10 (100)
Female	0 (0)	2 (18)	8 (44)	2 (17)	5 (45)	4 (100)	8 (100)	0 (0)
Stage, n (%)								
I	0 (0)	5 (45)	2 (11)	0 (0)	3 (27)	3 (75)	4 (50)	0 (0)
II	1 (8.3)	0 (0)	3 (17)	1 (8.3)	3 (27)	0 (0)	2 (25)	0 (0)
III	4 (33)	2 (18)	6 (33)	5 (42)	3 (27)	1 (25)	2 (25)	1 (10)
IV	7 (58)	4 (36)	7 (39)	6 (50)	2 (18)	0 (0)	0 (0)	9 (90)
cT, n (%)								
cT1 or cT2	1 (8.3)	5 (45)	6 (33)	0 (0)	6 (55)	3 (75)	6 (75)	0 (0)
cT3 or cT4	11 (92)	6 (55)	12 (67)	12 (100)	5 (45)	1 (25)	2 (25)	10 (100)
cN, n (%)								
cN0	1 (8.3)	4 (36)	6 (33)	5 (42)	10 (91)	4 (100)	7 (88)	1 (10)
cN1	11 (92)	3 (27)	8 (44)	7 (58)	1 (9)	0 (0)	1 (12)	9 (90)
cN2 or cN3	0 (0)	4 (36)	4 (22)	0 (0)	0 (0)	0 (0)	0 (0)	0 (0)
cM, n (%)								
cM0	5 (42)	7 (64)	10 (56)	6 (50)	9 (82)	4 (100)	8 (100)	2 (20)
cM1	7 (58)	4 (36)	8 (44)	6 (50)	2 (18)	0 (0)	0 (0)	8 (80)

**Table IV:** CTC detection by type of cancers.

	Esophagus (n=12)		Stomach (n=11)		Colon (n=18)		Pancreas (n=12)		Liver (n=11)		Endometrium (n=4)		Cervix (n=8)		Prostate (n=10)	
								
	CTC(+)	CTC(−)	CTC(+)	CTC(−)	CTC(+)	CTC(−)	CTC(+)	CTC(−)	CTC(+)	CTC(−)	CTC(+)	CTC(−)	CTC(+)	CTC(−)	CTC(+)	CTC(−)
All patients, n (%)	6 (50)	6 (50)	4 (36)	7 (64)	6 (33)	12 (67)	6 (50)	6 (50)	5 (45)	6 (55)	3 (75)	1 (25)	2 (25)	6 (75)	5 (50)	5 (50)
Stage, n (%)																
I	0 (0)	0 (0)	1 (9.1)	4 (36)	0 (0)	2 (11)	0 (0)	0 (0)	2 (18)	1 (9.1)	2 (50)	1 (25)	2 (25)	2 (25)	0 (0)	0 (0)
II	1 (8.3)	0 (0)	0 (0)	0 (0)	1 (5.6)	2 (11)	0 (0)	1 (8.3)	2 (18)	1 (9.1)	0 (0)	0 (0)	0 (0)	2 (25)	0 (0)	0 (0)
III	1 (8.3)	3 (25)	1 (9.1)	1 (9.1)	2 (11)	4 (22)	3 (25)	2 (17)	1 (9.1)	2 (18)	1 (25)	0 (0)	0 (0)	2 (25)	1 (10)	0 (0)
IV	4 (33)	3 (25)	2 (18)	2 (18)	3 (17)	4 (22)	3 (25)	3 (25)	0 (0)	2 (18)	0 (0)	0 (0)	0 (0)	0 (0)	4 (40)	5 (50)
cT, n (%)																
cT1 or cT2	1 (8.3)	0 (0)	2 (18)	4 (36)	2 (11)	4 (22)	0 (0)	0 (0)	4 (36)	2 (18)	2 (50)	1 (25)	2 (25)	4 (50)	0 (0)	0 (0)
cT3 or cT4	5 (42)	6 (50)	2 (18)	3 (27)	4 (22)	8 (44)	6 (50)	6 (50)	1 (9.1)	4 (36)	1 (25)	0 (0)	0 (0)	2 (25)	5 (50)	5 (50)
cN, n (%)																
cN0	1 (8.3)	0 (0)	1 (9.1)	3 (27)	1 (5.6)	5 (28)	4 (33)	1 (8.3)	0 (0)	1 (9.1)	3 (75)	1 (25)	2 (25)	5 (63)	1 (10)	0 (0)
cN2 or cN3	5 (42)	6 (50)	3 (27)	4 (36)	5 (28)	7 (39)	2 (17)	5 (42)	5 (45)	5 (45)	0 (0)	0 (0)	0 (0)	1 (13)	4 (40)	5 (50)
cM, n (%)																
cM0	2 (17)	3 (25)	2 (18)	5 (45)	3 (17)	7 (39)	3 (25)	3 (25)	5 (45)	4 (36)	3 (75)	1 (25)	2 (25)	6 (75)	1 (10)	1 (10)
cM1	4 (33)	3 (25)	2 (18)	2 (18)	3 (17)	5 (28)	3 (25)	3 (25)	0 (0)	2 (18)	0 (0)	0 (0)	0 (0)	0 (0)	4 (40)	4 (40)

CTC, circulating tumor cells.
